# Electrospinning Nanofibers as a Dressing to Treat Diabetic Wounds

**DOI:** 10.3390/pharmaceutics15041144

**Published:** 2023-04-04

**Authors:** Eun Jo Jang, Rajkumar Patel, Madhumita Patel

**Affiliations:** 1 Nano Science and Engineering, Integrated Science and Engineering Division (ISED), Underwood International College, Yonsei University, Songdogwahak-ro, Yeonsu-gu, Incheon 21983, Republic of Korea; 2 Energy & Environmental Science and Engineering (EESE), Integrated Science and Engineering Division (ISED), Underwood International College, Yonsei University, 85 Songdogwahak-ro, Yeonsu-gu, Incheon 21938, Republic of Korea; 3 Department of Chemistry and Nanoscience, Ewha Womans University, 52 Ewhayeodae-gil, Seodaemun-gu, Seoul 03760, Republic of Korea

**Keywords:** electrospun nanofiber, diabetic mellitus, wound healing, wound dressing, polymers

## Abstract

Globally, diabetic mellitus (DM) is a common metabolic disease that effectively inhibits insulin production, destroys pancreatic β cells, and consequently, promotes hyperglycemia. This disease causes complications, including slowed wound healing, risk of infection in wound areas, and development of chronic wounds all of which are significant sources of mortality. With an increasing number of people diagnosed with DM, the current method of wound healing does not meet the needs of patients with diabetes. The lack of antibacterial ability and the inability to sustainably deliver necessary factors to wound areas limit its use. To overcome this, a new method of creating wound dressings for diabetic patients was developed using an electrospinning methodology. The nanofiber membrane mimics the extracellular matrix with its unique structure and functionality, owing to which it can store and deliver active substances that greatly aid in diabetic wound healing. In this review, we discuss several polymers used to create nanofiber membranes and their effectiveness in the treatment of diabetic wounds.

## 1. Introduction

Diabetes mellitus (DM) is one of the most diagnosed metabolic diseases that predominately causes hyperglycemia [1,2]. Without proper treatment, diabetes causes many complications, such as slowed wound healing and the development of ulcers, resulting in increased morbidity and mortality. Wound sites create a breeding ground for various bacteria, due to their high levels of glucose, and exhibit decreased neovascularization and consequently, impaired wound healing [3].

Wound dressings are crucial for the healing of diabetic wounds. Various dressings can protect the wound area from external risk factors and provide a suitable environment for accelerated wound healing [4]. To provide the wound area with the most benefits, an ideal wound dressing should have the ability to absorb exudate, protect the wound from infection, maintain the moisture level, facilitate gas exchange, remain biocompatible and degradable, promote tissue regeneration, and be easy to remove from the wound [5,6]. Currently, many modern wound dressings are available in the market; however, they all have their own limitations. Wound dressings, such as gauze, are inexpensive but can damage the wound site upon removal [7]. Semi-permeable films permit gas exchange, although they have a low absorption capacity [8]. In contrast, foam dressings possess a larger absorption capacity; however, their long residence time leads to dryness of the wound area [9]. Furthermore, although hydrogel dressings are capable of maintaining a moist microenvironment surrounding the wound, they can increase the risk of bacterial infection [9,10]. As a result, it is imperative to develop effective dressings capable of sustainable medication release for improving diabetic wound care. Recently, many efforts have been made to create better dressings, including the addition of nanofiber membranes [11,12,13,14]. These wound dressings have shown efficacy in treating diabetic wounds, promoting tissue and vascular regeneration, and accelerating wound closure [15,16].

### 1.1. Nanofiber Dressing

**Figure 1:**
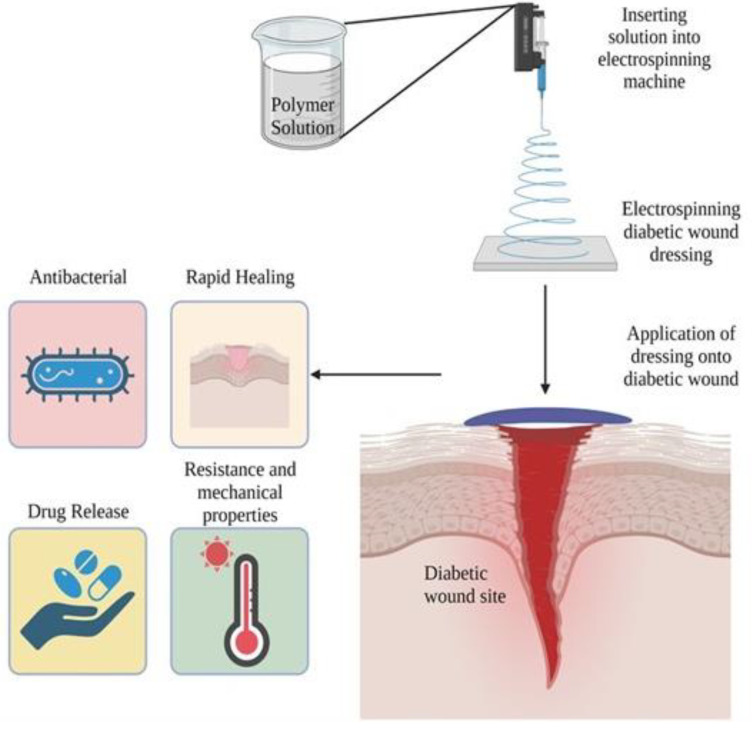
Schematic diagram showing electrospun nanofibers for diabetic wound healing. Created with BioRender.com.

**Figure 2:**
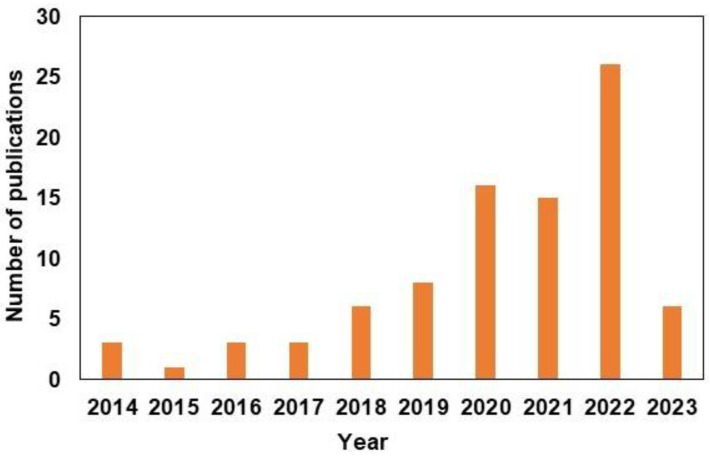
The number of articles published in the field of “Electrospinning nanofiber for diabetic wound healing” (source Scopus).

Wound dressings made of nanofibers have been developed predominantly through electrospinning [17], blow spinning [18], microfluidic spinning [19], and centrifugal spinning techniques [20]. Of these techniques, electrostatic spinning is the most widely used because of its ability to create diameter ranges of the order of microns or nanometers, which have noticeable benefits [21]. Nanofiber wound dressings prepared via electrospinning have several advantages. First, the developed structure and function is similar to the innate extracellular matrix (ECM), which creates the ideal environment for adhesion, proliferation, migration, and differentiation of cells [22,23]. Second, the created matrix can incorporate the biocompatibility of natural polymers along with the enhanced mechanical properties that synthetic polymers provide [24]. Furthermore, its large surface area and porous structure facilitate loading of various bioactive molecules, which can be sustainably released into the wound area. The rate at which molecules are released from dressings can be controlled by modifying the structure and size of the pores, thereby accelerating wound healing [25]. Hence, electrospun nanofibers are a great alternative to the current methods of diabetic wound treatment, owing to their various benefits (Figure 1). To better understand the current state of research on the use of electrospun nanofiber membranes for diabetic wound healing, we searched Scopus for “electrospinning nanofiber for diabetic wound healing”. The search results are shown in Figure 2. The number of articles published on this topic has increased, indicating that wound healing in diabetes has attracted widespread attention. In the last few years, several reviews have been published on this topic [26,27,28]. Liu et al. and Yan et al. reviewed the development of electrospinning technology, and Gao et al. summarized bioactive compound loaded nanofibers for diabetic wound healing. In this review, we discussed the common polymers used to fabricate electrospun nanofibers, particularly for diabetic wound healing. We summarized the latest developments in diabetic wound healing, which can offer new ideas and insights into the improved treatment of diabetic wounds.

### 1.2. Diabetic Wounds

Diabetic wounds are severe injuries which are common in patients with diabetes. These wounds are chronic, susceptible to infection, and require extended repair time. The healing process is delayed by various internal factors such as microvascular dysfunction, peripheral neuropathy, and hypoxia, which disrupt the normal healing phases of hemostasis, inflammation, proliferation, and remodeling [29,30]. Peripheral neuropathy, the most common type of diabetic neuropathy, often damages nerve tissue. As a result of diabetic peripheral neuropathy, wound healing is delayed due to sensory, motor, and autonomic dysfunction. Sensory neuropathy leads to a loss or absence of pain sensation, while autonomic neuropathy affects skin temperature, sweat production, and blood flow in the foot [30,31].

Additionally, diabetic wounds have increased levels of reactive oxygen species (ROS), matrix metalloproteinases (MMPs), and pro-inflammatory cytokines [including tumor necrosis factor-α (TNF-α) and Interleukin-6 (IL-6)], in comparison with nondiabetic wounds [32,33]. As a result of excess inflammatory factors and active substances such as cytokines, growth factors, and antibiotics, the ECM deteriorates rapidly, delaying stages such as re-epithelialization and disrupting diabetic wound healing. Diabetic wound healing times may also be prolonged because pro-inflammatory macrophage “M1” cannot be converted to the healing-promoting macrophage “M2”, which reduces the expression of anti-inflammatory cytokines (IL-10) [34].

## 2. Polymeric Nanofibers for Wound Dressing

Wound dressings are essential for achieving better and faster wound closures [35]. Electrospinning is predominantly used in the development of dressing materials because of its simplicity and flexibility in creating polymers from different materials. Polymers are broadly divided into two categories: synthetic and natural. Natural polymers exhibit better biocompatibility, degradation, and lower immune responses, whereas synthetic polymers exhibit excellent mechanical strength, stiffness, and flexibility. Blending these polymers is advisable to obtain maximum benefits.

### 2.1. Synthetic Polymers

Synthetic polymers are artificially synthesized and customized to obtain essential properties. Their electrospun fibers are mechanically strong and stable, and they can be directly applied for diabetic wound treatment. Table 1 lists the synthetic polymers commonly used for electrospun fibers to improve diabetic wound healing.

#### 2.1.1. Poly (ε-caprolactone) (PCL)

PCL is a widely used biocompatible and degradable polymer that minimizes environmental impact upon degradation. PCL is an FDA approved material that is applied in wound dressings due to its outstanding spinnability and mechanical strength. Chen et.al. fabricated PCL electrospun nanofiber scaffolds with custom 3D shapes, and either radial or vertical organizations. Bone marrow-derived mesenchymal stem cells (BMSCs) laden on the nanofibers facilitate tissue development, angiogenesis, and enhanced collagen (Col1) deposition. It can also switch immune responses to help generate tissue by inhibiting the formation of M1 macrophages and pro-inflammatory cytokines while activating M2 macrophages, leading to anti-inflammatory cytokine production. This membrane promotes the immune system and facilitates wound healing [36]. The use of ECM biomimetic structures has been of constant interest for diabetic wound healing. Using ECM components Col, PCL, and bioactive glass nanoparticles, Gao et al. developed a scaffold (CPB) that could aid in endothelial cell proliferation and angiogenesis in vivo. The scaffold showed notable improvements in the expression of angiogenic factors (VEGF, Hif-1α, Col I, and α-SMA) and Col deposition compared with control group. Additionally, the scaffold improved the differentiation process and accelerated wound healing [37]. Ways to inhibit the effects of hypoxia-inducible factor 1 α (HIF-1α) are being investigated to promote diabetic wound healing. Releasing dimethyloxalylglycine (DMOG), a competitive inhibitor of prolyl hydroxylases (PHDs), near the wound area can readily stabilize HIF-1α and accelerate wound healing. The sustained release of DMOG from PCL/type I collagen (Col I) electrospun core-sheath fibers enhanced the migration and expression of wound healing genes in vitro. Furthermore, DMOG stabilizes HIF-1α levels and improves the healing process in vivo [38]. The PCL mesh with DMOG additionally enhanced the expression of growth factors (e.g., IGF-1, HB-EGF, and NGF) and anti-inflammatory factors (e.g., transforming growth factor (TGF)-β1 and IL-4) while decreasing expression levels of pro-inflammatory factors (e.g., IL-1β and IL-6) in vitro. The mesh increased the wound closure rate, re-epithelialization, maturation, and deposition of Col in vivo, two weeks after implantation [39].

A hybrid scaffold of polyurethane (PU) and PCL (PU/PCL) showed potential as a wound dressing material for diabetic wound areas. The dressing showed great permeability to oxygen and had a good degree of swelling and water content; its biocompatibility towards L-929 cells was assessed to be 86.9% when applied. Combined with its low cost, the PU/PCL dressing is an alternative to existing dressing materials for diabetic wound healing [40]. Loading curcumin (Cur) into the matrix of PCL/gum tragacanth (GT) (PCL/GT/Cur) nanofibers has been shown to accelerate the healing process of diabetic wounds, in addition to having the ability to remain resistant against *Staphylococcus aureus* and spectrum of β –lactamase. In vivo application of PCL/GT/Cur to diabetic wounds resulted in excellent granulation and cellular proliferation, Col deposition, and re-epithelization of the tissue compared with the control wounds. The developed scaffold showed an increase in angiogenesis, granulation of the tissue area, a number of fibroblasts, and a decreased epithelial gap [41]. Similarly, loading bixin with PCL nanofibers showed an improvement over innate PCL nanofiber dressings. In vitro assessment of the nanofibers showed that bixin could be released from the matrix over a two-week period, showing its sustained drug release ability. An in vivo study in diabetic mice showed that bixin plays a successful role in accelerating the wound-healing process and reducing scar tissue [60]. Calreticulin (CRT), an endoplasmic reticulum chaperone protein, enhances wound healing. In a study by Stack et al., CRT incorporated within the PCL/Col 1 nanofibers achieved a sustained release, which stimulated faster keratinocyte migration and cell polarization in vitro. In addition, the scaffold upregulated TGF-β1 protein and resulted in remarkable mechanical properties and integrity, with protection against degradation exhibiting therapeutic potential against diabetic foot ulcers [42]. In another study, glutathione, a biomolecule immobilized on PCL meshes, reduced wound-healing times in diabetic rats. Sustained release of glutathione from the mesh for up to 20 days exhibited antibacterial activity and showed its potential to be used as a wound healing material for diabetic patients [61]. A three-layered scaffold built with PCL, PCL/Col, and Col nanofibers could hold the *Melilotus officinalis* extract within its matrix. The inner Col layer was able to absorb wound exudates and promote angiogenesis, the PCL-Col middle layer improved the mechanical strength, and the outer layer protected the wound from contamination. The extract-loaded dressings exhibited proper re-epithelialization of diabetic wounds and Col production and deposition in the newly formed skin [43]. Metformin hydrochloride (MH) loaded within hyaluronic acid (HA)-keratin-polyethylene oxide and PCL nanofiber can be used to treat diabetic wounds. The sustained release of MH for over 20 days prolonged its bioactivity near diabetic wounds. The nanofibers showed excellent biocompatibility and notable mechanical and chemical properties that assisted in diabetic wound healing [62].

**Figure 3:**
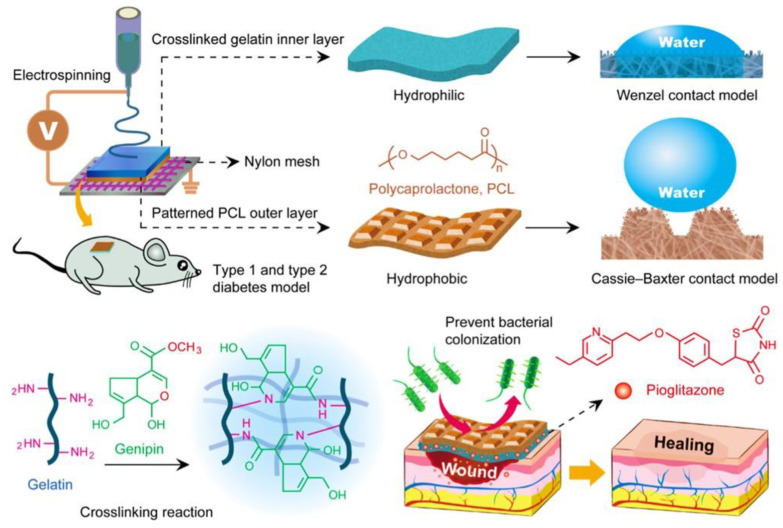
Graphical representation of the asymmetric wettable preparation for wound dressings. Reproduced with permission from Ref. [44]. Copyright 2020, American Chemical Society.

The organization of fibers or scaffolds remains an area of research, as their orientation has been shown to play a role in the effectiveness of diabetic wound treatments. In a study by Sun et al., nanofibrous scaffolds of PCL/Col1 with basketweave-like patterns mimicked the natural Col arrangement. Compared with aligned or randomly oriented nanofibrous scaffolds, the basketweave patterns showed the greatest improvements and enhancements in their ability to heal wounds in diabetic rats. The patterns also accelerated fibroblast migration and promoted angiogenesis, and the inflammation levels noticeably decreased [63]. The use of asymmetric wettable dressings to treat diabetic wounds is another promising approach. An asymmetric wettable dressing developed by Yu et al. using PCL by electrospinning pioglitazone-incorporated gelatin (Gel-pio) to form a hydrophobic outer layer while creating a hydrophilic layer on the inside. The outer layer acted as a water barrier for the wound area to remain dehydrated, whereas the inner layer protected against bacterial growth or infection. The in vitro results showed that gelatin (GEL) nanofibers incorporated with a pioglitazone accelerated cell proliferation and migration. In vivo, the composite dressings enhanced diabetic wound healing by enhancing cell proliferation and angiogenesis, while modulating the expression of VEGF, MIP-2, IL-1β, IL-6, TNF-α, MMP-9, and TGF-β (Figure 3) [44].

#### 2.1.2. Poly(lactic-co-glycolic acid) (PLGA)

PLGA, a copolymer of poly (L-lactic acid (PLA) and polyglycolic acid (PGA), has a low toxicity toward humans. PLGA is another attractive biocompatible polymer because it is FDA approved and is used in the development of nanofiber matrices. [64]. PLGA has been used in various hybrid scaffolds for diabetic wound treatments [65].

**Figure 4:**
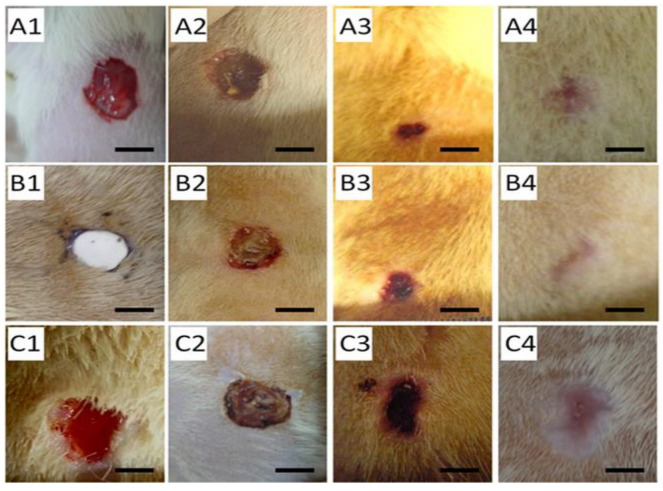
Wound healing on day 0 (1), 3 (2), 7 (3), and 14 (4) post treatment: (**A**) the PLGA with metformin group; (**B**) virgin PLGA; (**C**) the conventional gauze sponge group. Scale bar = 5 mm. Reproduced with permission from Ref. [66]. Copyright 2014, American Chemical Society.

Utilization of Col/poly-D-L-lactide-glycolide (PLGA) nanofibers to release of glucophages is a potential diabetic wound treatment approach. The PLGA/Col membrane released glucophages into the wound area over a three-week period. In addition to its sustainable drug release ability, increasing the amount of retained water promoted effective healing of diabetic wounds, enhanced Col content, and induced faster wound healing [45]. Furthermore, PLGA-Col nanofibrous scaffolds could hold and sustainably release recombinant human platelet-derived growth factor (rhPDGF) to promote diabetic wound healing. The scaffold exhibited a remarkable water retaining capacity while allowing more Col to form near the damaged area. In addition, the scaffold promoted re-epithelialization of the wound, which allowed the wound to heal at a faster rate and decreased the healing period [65]. New methods of aiding wound healing in patients with diabetes are continuously being developed. Nanofibrous PLGA scaffolds incorporating antibiotics such as vancomycin and gentamicin, and platelet-derived growth factor (PDGF) accelerated wound healing. The release of PDGF and antibiotics allowed the wound area to clear any infections and accelerated the healing process. The membrane induced higher expression of the angiogenesis marker CD31, indicating that the film could accelerate the growth of new tissue around the area [46]. Similarly, Lee et al. developed a method to deliver metformin (Met) by incorporating it into the wound dressing of PLGA nanofibers. The nanofiber scaffold that contained Met within its matrix could be released over a three-week period and had greater hydrophilicity, while also having better water retaining abilities than regular PLGA fibers. In combination with the drug release system, an in vivo application of PLGA with Met resulted in an enhancement in diabetic wound healing and helped re-epithelialize the wound area (Figure 4) [66]. In another study, the impregnation of epigallocatechin-3-O-gallate (EGCG) into an HA-PLGA scaffold released EGCG over four weeks, demonstrating its ability to treat wounds in the long term. The HA/PLGA/EGCG membrane showed an increased number of fibroblasts that were able to attach to the matrices compared to the HA/PLGA membrane. Moreover, the scaffold showed greater Col deposition, re-epithelialization, and neovascularization than the control and HA/PLGA membranes [47]. In a study by John et al., a PLGA/GEL fiber aerogel containing the LL-37-mimic peptide W379 enhanced proliferation and migration of keratinocytes and fibroblasts, while the introduction of macrochannels within the aerogel improved cell penetration. Moreover, the patterned macrochannels together with the incorporation of W379 greatly increased the healing time of diabetic wounds through re-epithelization by upregulating p38 mitogen-activated protein kinase expression [48]. Membranes from living cells have attracted attention as a new way of improving the bioactivity of living materials. A PLGA scaffold along with lipopolysaccharide/interferon-γ, an activated macrophage cell membrane, enhanced proliferation and migration of keratinocytes while speeding up wound closure, re-epithelialization, and angiogenesis when loaded with BMMSCs (RCM-fiber-BMMSCs). In addition, the scaffolds upregulated the expression of 449 genes related to wound healing compared to the nanofibers without cell membranes [67].

#### 2.1.3. Poly (L-lactic acid) (PLA)

**Figure 5:**
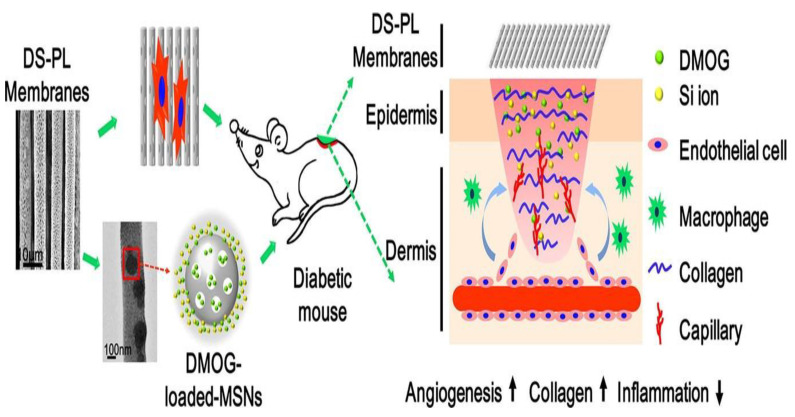
Graphical representation of electrospun membranes containing DMOG-loaded mesoporous silica nanoparticles (DS) for diabetic wound healing. Reproduced with permission from Ref. [49]. Copyright 2014, Elsevier.

PLA has excellent mechanical properties, biocompatibility, and degradability [68]. PLA has shown potential for use in biomedical applications such as wound healing, drug delivery, and tissue engineering, and it has been approved by the FDA [69]. Recently, it was shown that electrospun PLA scaffolds could be used as alternatives to promote cell migration and proliferation, and Col formation [70]. Alignment of porous poly-L-lactide (PLLA) membranes with DMOG loaded mesoporous silica nanoparticles (DS) enhanced angiogenesis in diabetic wounds. The fibers were oriented toward a single direction and contained nano-pores that could contain DMOG and Si ions. These porous characteristics allow for the controlled release of the drug contained within the fibrous matrix. The DS-PL membrane enhanced proliferation, migration, and angiogenesis in diabetic wounds, while also improving vascularization and epithelialization of the wounds. The fibers were able to control inflammation levels, which could further improve wound healing (Figure 5) [49].

**Figure 6:**
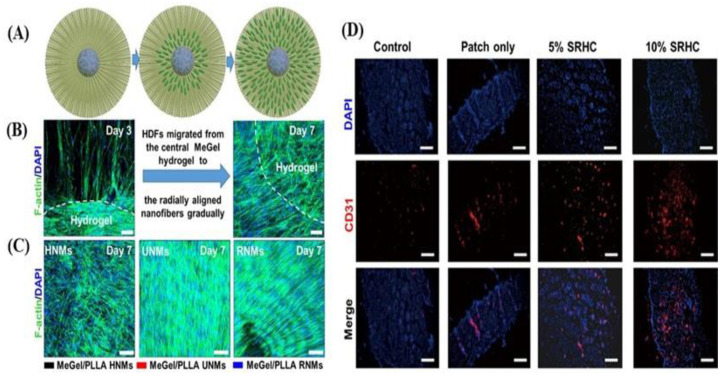
(**A**) Graphical representation of the cell migration process of HDFs on the MeGel/PLLA RNMs. (**B**) Fluorescent images of HDFs on the MeGel/PLLA radially oriented nanofiber mats (RNMs) at day 3 and 7. (**C**) Fluorescent images of HDFs on MeGel/PLLA uniaxially oriented nanofiber mats (UNM), haphazardly oriented nanofiber mats (HNM), and RNMs on day 7. Scale bars = 100 μm. (**D**) Fluorescent images of the regenerated skin tissue stained with CD31 (red) and DAPI (blue) 18 days post-surgery. Scale bars = 50 μm. Reproduced with permission from Ref. [50]. Copyright 2022, Elsevier.

The orientation of the electrospun nanofibers significantly affected their mechanical properties. Wu et al. designed electrospun methacrylated gelatine (MeGel)/PLLA radially oriented nanofiber mats (RNMs) as dressing patches. Dressing patches, in combination with *Salvia miltiorrhiza Bunge-Radix puerariae* (SRHC), a herbal compound, showed improvements in the closure and healing of diabetic wounds, as they promoted the proliferation and migration of human dermal fibroblasts (HDFs) compared to simple medical gauzes. When loaded with SRHC, the dressings exhibited excellent hemostatic performance, and they presented antibacterial properties against *Escherichia.coli* and *S. aureus.* Furthermore, the 10% SRHC-loaded dressing reduced inflammation and promoted vascularization and regeneration of hair follicles (Figure 6) [50].

The utilization of NIR-assisted oxygen delivery along with black phosphorus (BP) nanosheets, hemoglobin (Hb), and PLLA has been discussed as a new way of dealing with harsh hypoxic microenvironments (HMEs) in diabetic wounds. When BP is exposed to NIR radiation, it can generate heat, allowing Hb to release oxygen into the wound site. This offers antibacterial activity along with accelerated wound healing phases in both angiogenesis and cell migration. The wound area exhibited antibacterial activity against methilicillin-resistant *S. aureus and* E. coli, while exhibiting great mechanical strength to support its use in diabetic wound healing [71]. Furthermore, PLLA nanofibers promoted skin immunity, neutrophil migration, and fibroblast regulation by releasing GM18. The dressing was able to release GM18 over seven days, enabling controlled and sustained release of GM18. The released GM18 targeted the integrin α4β1, which inhibited EDA-fibronectin- α4β1 binding and promoted signaling of the ERK1/2 pathways, leading to improvement of the healing process. The controlled release of such substances can achieve dressing longevity and accelerate the wound healing process [72].

To aid tissue formation in patients with diabetes, the combination of connective tissue growth factor (CTGF) in porous shell fibers (PLA-PVA) achieved better wound healing. These scaffolds facilitated rapid cell proliferation, migration, and angiogenesis. The release of CTGF from the porous shell fibers allowed cells in the wound area to receive CTGF over a long period [73]. In another study, PLA electrospun with HA, valsartan, and ascorbic acid was found to be an effective scaffold for diabetic wounds. The mechanical properties of the nanofibers closely matched those of the human skin. Moreover, the porous scaffold exhibited high drug solubility, oxygen permeability, and fluid uptake, and sustainably released valsartan from matrix. Compared with the traditional and existing methods of treating diabetic wounds, the release of valsartan from its nanofiber scaffold showed a much greater re-epithelization rate of up to 85.5%. Overall, the incorporation of valsartan allowed for much faster healing of the wound area, whereas the PLA/HA nanofibrous scaffolding provided a release mechanism and protection from the outside [51]. Cerium oxide nanoparticles (nCeO_2_), which are ROS scavengers within a trilayer membrane of PLA/PVA/PLA, mimic the nature of ECM. The controlled release of nCeO_2_ enhances diabetic wound healing by increasing cell adhesion, growth, and proliferation. In addition, the mechanical, thermal, and morphological properties were enhanced by the introduction of nCeO_2_ [52]. Wound dressings, along with their antibacterial properties, improved diabetic wound healing, which was achieved through the incorporation of selenium (Se), graphene oxide (GO), and clarithromycin in PLA nanofibers. The mesh enhanced the healing capabilities by promoting cell adhesion and migration while being mechanically and chemically stable. Furthermore, their antibacterial properties are beneficial for the potential use of dressings [74]. Similarly, PLA/GEL nanofiber scaffolds showed antibacterial properties when combined with epidermal growth factor (EGF). The scaffold was effective in combating *E. Coli* and *S. aureus* infections. In addition, the dressings exhibited excellent mechanical properties, including maximum elongation, tensile strength, and tensile modulus. Furthermore, their ability to promote the proliferation of L929 fibroblasts cells assisted in diabetic wound healing and re-epithelization [53].

#### 2.1.4. Polyvinyl Alcohol (PVA)

PVA is soluble in water, easy to manufacture, biocompatible, and degradable, and has excellent mechanical and chemical properties [75]. Its solubility in water provides an easy way to process and cross-link polymers; it is being investigated as an alternative for creating diabetic wound dressings.

**Figure 7:**
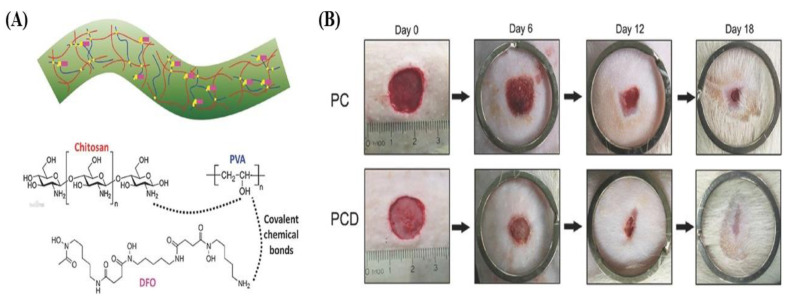
Hydrogel electrospun fibers’ structure and the covalent chemical bonds among CS, PVA, and DFO drug (**A**), photographs of the wound healing at days 0, 6, 12, and 18 post-treatment (**B**). Reproduced with permission from Ref. [54]. Copyright 2016, Wiley.

To further enhance the properties of PVA and increase its efficiency in diabetic wound treatment, it would be beneficial to incorporate it into various other natural polymers. For example, mats made of PVA, in addition to non-mulberry SF (NMSF), growth factors, and LL-37 antimicrobial peptides, showed greater healing properties than mulberry SF-based dressings. NMSF-based dressings exhibited faster tissue development, enhanced angiogenesis, re-epithelization, and Col deposition in the wound area. The results suggested the potential for application of NMSF-based dressings in diabetic wounds [76]. A new way of putting deferrioxamine (DFO) into the PVA matrix of hydrogel nanofibrous scaffolds is to further improve the angiogenesis of wound areas in diabetic patients. The hydrogel scaffold held and sustainably released DFO for up to 72 h, thereby accelerating the initial healing process. These scaffolds increased neovascularization and promoted cell proliferation and vessel formation. In addition, the scaffolds upregulated Hif-1α and vascular endothelial growth factor expression to enhance wound healing. Usage of DFO with hydrogel nanofibrous scaffolds show its ability to inhibit prolyl-hydroxylase cofactors while upregulating the expression of Hif1-α, significantly helping the wound healing process (Figure 7) [54]. Similarly, scaffolds with GT, PCL, and PVA have the potential to be used as dressing materials for diabetic wounds. PCL/GT/PVA showed acceptable tensile strength and Young’s modulus of 2.7 and 5.6 MPa, respectively, and exhibited the ability to attach to cell tissues. This allows for successful and fast proliferation of cells, and experiments on diabetic rats have shown increased epithelization and wound repair [55].

Another nanofiber mat made of PLA along with PVA, MH, and fish sarcoplasmic protein (FSP) exhibited decreased crystallinity. The ability of nanofibers to sustainably release drugs enhanced diabetic wound healing. The nanofibers exhibited properties suitable for the adhesion and proliferation of HaCaT cells in vitro [77]. Propolis is commonly used for treating inflammatory dermal diseases. Alberti et al. incorporated propolis into a PVA scaffold that showed excellent wound treatment capabilities and no cytotoxicity in fibroblasts. Comparing untreated and treated wounds, PVA and propolis showed the highest wound closure rate at 68% after seven days. Its ability to continuously release propolis into the wound area allowed for faster treatment and healing of the wound area [78].

Due to its biodegradability, biocompatibility, antimicrobial properties, cellular binding ability, and wound healing effects, Chitosan (CS), a polysaccharide, is often blended with PVA [79]. Majd et al. designed a wound dressing made of PVA and CS, which allowed for a high moisture transmission rate and antimicrobial activity that stimulated the wound healing process. In addition, the dressing exhibited an excellent odor inhibition capacity and no noticeable cytotoxicity. In an in vivo study on diabetic rats, the dressings showed accelerated wound healing capabilities [56]. Furthermore, a wound dressing with a combination of ursolic acid (UA) and CS/PVA exhibited excellent hydrophilicity and wettability, and reduced inflammation around the wound area. The systematic release of UA helps with wound treatment, decreasing the release levels of TNF-α and IL-6 inflammatory factors. It improves the wound closure rate, promotes revascularization and re-epithelialization, and helps to regenerate hair follicles [80]. Similarly, the incorporation of zinc oxide (ZnO) within the matrix of PVA and CS allowed for the development of effective diabetic wound dressings. The sustained release of ZnO into the wound area resulted in antibacterial properties against *E. coli*, *P. aeruginosa*, *Bacillus subtilis*, and *S. aureus*, which was an improvement over the innate CS/PVA nanofiber membrane. Furthermore, the higher antioxidant properties of mats increased the performance of wound contraction within a time interval of 12 days [57]. A PVA nanofiber wound dressing combined with CS and anemoside B4 (ANE) also promoted rapid closure of the diabetic wound, accelerating re-epithelization and deposition of the Col matrix. The nanofiber matrix incorporating ANE was able to inhibit inflammatory responses and facilitated reduction of ROS generation and cytokine release (TNF-α and IL-6) both in vitro and in vivo. Additionally, the nanofiber matrix showed better water absorption, mechanical properties, and hemostatic properties [81]. Enteromorpha polysaccharide (EPP), which has antibacterial and antioxidant activities when blended with PVA, improves the repair process of skin wounds in diabetic mice. The dressing inhibits the inflammatory response while accelerating the healing process. While remaining biocompatible, the dressing achieved a high water absorption capacity, demonstrating its potential for use as a diabetic wound dressing [82].

Natural products play important roles in wound healing. The antibacterial properties of Cur derivatives (CD) in PVA nanofibers were effective against *S. aureus,* thereby preventing potential infections around the diabetic wound area. CD enhanced wound healing by activating glycogen synthase kinase-3 beta and TGF-β1 kinase domain signaling pathways [83]. PVA nanofibers containing neomycin sulfate (NS) and *Malva sylvestris* extract (MS) exhibited excellent water uptake and tensile strength, while releasing NS for 60 h and MS for 84 h. The sustained release of MS exerted antibacterial activity against *S. aureus* and *E. coli,* leading to a diabetic wound healing rate of 96.08% by day 14 in vivo [84]. Astragalus polysaccharide (APS) and astragaloside IV (AS) with polyvinyl alcohol (PVA) dressings have been developed to treat diabetic foot ulcers. The matrix facilitates sustained release of molecules and the subsequent inhibition of wound inflammation. Moreover, the dressing helped create an environment for faster deposition of Col fibers and re-epithelialization in diabetic rat wounds [85].

#### 2.1.5. Polyvinylpyrrolidone (PVP)

PVP is a water-soluble and biocompatible polymer with good solubility in various organic solvents, making it suitable for developing nanofibers through electrospinning. The addition of gentiopicroside (GPS), thymoquinone (TQ), and methyl ether polyethylene glycol (m-PEG) to the PVP nanofiber matrix accelerated diabetic wound healing. The nanofiber mats exhibited antibacterial properties against P. aeruginosa, which is a problematic bacterium for wound treatment, while also exhibiting anti-inflammatory properties [58]. Similarly, the sustained release of pioglitazone (PGZ), an antidiabetic drug, from PVP fibers accelerated diabetic wound healing and showed anti-inflammatory properties. The released PGZ also showed a high retention rate on the skin, minimizing contact with the blood circulation [59]. Similarly, pioglitazone hydrochloride (PHR)-loaded PVP/PCL fibers increased fibroblast proliferation and epidermal regeneration while reducing oedema, neutrophil infiltration, and inflammation. The fibrous mat successfully accelerated diabetic wound healing both in vitro and in vivo [86]. To overcome the limited water absorption of conventional dressings, a combination of gelation fabrics were used as a hydrophilic layer, and polyvinyl butyral (PVB) along with PVP were used as a hydrophobic layer to create a double-layered dressing that pumps the exudate from the wound area. This leads to a low inflammatory response and moisturization of the wound area, improving the wound healing quality and accelerating the healing process [87].

### 2.2. Natural Polymers

Natural polymers have excellent biocompatibility and low immune responses. However, these materials exhibit poor mechanical properties. To overcome this disadvantage, natural polymers can be blended with synthetic polymers to greatly expand their applications. Table 2 lists the natural polymers that are widely used to create electrospun fibers that improve diabetic wound healing.

### 2.3. Gelatin(GEL)

GEL shows great potential as a diabetic wound dressing, owing to its various properties. It exhibits excellent mechanical and biological properties, allows structural modification, and is biocompatible and degradable [94]. Furthermore, GEL from Col has ECM-like properties and is known to enhance epithelialization and tissue formation, thereby being beneficial for diabetic wound treatment [95]. Despite these properties, GEL alone is not sufficient as a dressing material, owing to its poor compatibility with electrospinning processes that creates poor-quality nanofibers [96]. Arabinoxylan ferulate (AXF) and GEL at different ratios were impregnated with silver to demonstrate sustained drug release and antibacterial properties. Overall, the 4:1 ratio of GEL to AXF showed the best mechanical properties, and its porosity was the highest at 95.6%, showing successful release of silver over a period. To evaluate the antibacterial properties of silver, the membrane was tested against bacteria such as *P. aeruginosa, S. aureus,* and *Enterococus faecalis* to demonstrate its antibacterial activity against both gram-negative and gram-positive bacteria. Despite its mechanical and antibacterial properties, researchers have noted that this form of treatment should only serve as a secondary wound matrix rather than a primary treatment. The GEL-AXF mats degraded in aqueous media because of the instability of GEL/AXF in aqueous environments, which limited their long-term usage as a primary wound dressing [88]. Developing protein-based dressings is important for the treatment of diabetic wounds, as they are unlikely to cause an immunologic response and are relatively biocompatible. Cam et al. developed a dressing composed of a combination of GEL and bacterial cellulose (BC) to create a natural polymer scaffold. The combined GEL/BC polymers were further electrospun with Met or glybenclamide (Gb) to evaluate their ability to increase cell proliferation. When compared side-to-side, the GEL-BC-Gb system showed a greater healing effect on the wound than the GEL-BC-Met system. Successful re-epithelization and granulation of the tissue were observed in wounds treated with the GEL-BC-Gb compared to those treated with the Gel-BC-Met system. Overall, the sustained release of Gb and Met from wound dressings showed their ability to accelerate the healing process of diabetic wounds, whereas the GEL-BC polymer exhibited its ability to release drugs and remain biocompatible with minimal side effects [89].

**Figure 8:**
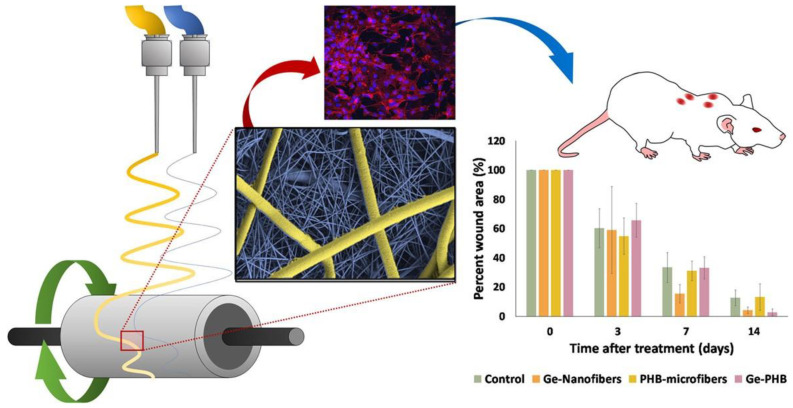
Graphical representation of PHB and Ge nanofibers as wound dressings. Reproduced with permission from Ref. [90]. Copyright 2021, Elsevier.

A scaffold developed with poly-3-hydroxybutyrate (PHB) microfibers and GEL nanofibers showed excellent characteristics in wound healing, promoting fibroblast assessment and tissue creation. The membrane also disallowed skin contraction and increased the healing rate while aiding hypodermis formation. The PHB-microfibers helped in linking the GEL nanofibers with each other, while GEL nanofibers worked as a reinforcing material to stop the membrane from shrinking. These synergistic effects of both fibers allowed GEL-PHB fibers to be successful in healing diabetic wounds (Figure 8). Mice treated with these fibers had a higher number of hair follicles and sweat glands while having a lower content of fibroblasts [90]. The development of two nanofiber series from polyurethane (PU) with cinnamon essential oil (CEO) and polyvinyl alcohol-gelatin (PVA/GEL) with nanoceria (nCeO_2_) resulted in improved mechanical and thermal properties. The nanofibers increased the cell population when used on a diabetic wound while inhibiting the growth of *S. aureus* and *E. coil* [97].

## 3. Silk

Silk fibroin is a natural polymer obtained from silkworm cocoons (*Bombyx mori*). It has been used in various areas of tissue regeneration, drug delivery and biomedical applications due to its excellent mechanical properties, biocompatibility, degradability, and nontoxicity.

**Figure 9:**
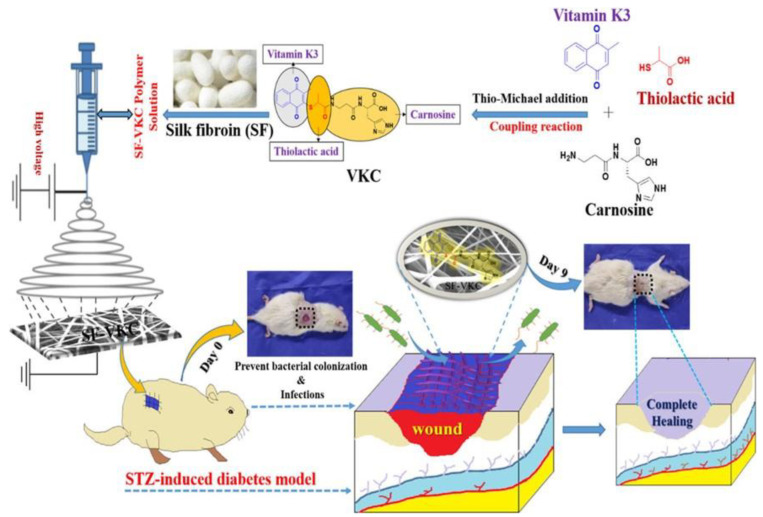
Schematic representation of the SF and VKC antibacterial agents used in electrospun fiber fabrication for STZ-induced diabetic wound healing applications. Reproduced with permission from Ref. [99]. Copyright 2021, American Chemical Society.

The incorporation Cur into the matrix of electrospun SF, PCL, and PVA nanofibers shows its potential for use as a diabetic wound dressing. The scaffold could sustainably release Cur into the wound area over several hours, and its excellent mechanical properties, such as tensile strength, made it an acceptable material for use as a wound dressing. Cur acted as both an antioxidant and anti-inflammatory substance in the wound area and accelerated the healing process. Furthermore, experiments on diabetic mice showed its rapid wound healing properties compared to traditional and existing healing methods [98]. The utilization of bioactive molecules in the dressings of diabetic wounds has been extensively investigated. To effectively control potential infection in the area, the use of vitamin K3 carnosine peptide (VKC) with a laden silk fibroin scaffold (SF-VKC) showed excellent cell viability and biocompatibility while exhibiting antibacterial properties against *S.aureus, E.coli,* and *P. aeruginosa*, showing that it is effective against both gram-positive and gram-negative bacteria. Furthermore, these dressings exhibited significant mechanical properties, including drug release, water uptake, and adhesiveness, while being relatively inexpensive to produce. The overall characteristics of the scaffolding helped increase the speed of cell migration and repair of diabetic wounds, showing its great potential as a wound-treating dressing material (Figure 9) [99].

A combination of Huangbai Liniment (Compound Phellodendron Liquid, CPL) with SF and poly-(L-lactide-co-caprolactone) (PLCL) was observed to aid in diabetic wound healing. With the sustained release of the drug over a long period, the bandages were able to achieve antimicrobial activity against *S. aureus* and *E. coli*. In addition, cell proliferation improved and expression levels of TGF-β signaling pathway components and Col increased. Through animal experimentation, these bandages ensured that the membrane worked as intended and inhibited inflammatory factors [91]. SF nanofibers, menstrual blood-derived mesenchymal stem cells (MenSCs), and human amniotic membrane (AM) used as bilayer scaffolding (bSC) showed potential for use as a diabetic wound dressings. The dressings exhibited the highest epidermal and dermal regeneration rates when bSC was combined with MenSCs. Furthermore, they facilitated the upregulation of expression of genes that are critically associated with wound healing compared to the expression of genes associated with the innate AM and bSC scaffolds [100]. Similarly, an SF nanofiber matrix containing hydroxyapatite (HAp) and Cur was effective in treating diabetic wounds. The SF/HAp-Cur nanofibers exhibited much better mechanical and water uptake properties than the innate SF/HAp nanofibers. Moreover, the sustained release of Cur into the wound area exerted its antibacterial properties against *S. aureus* and *E. coli* in addition to its healing action. The dressing achieved a wound closure rate of 99.6% on day 21, whereas the innate SF/HAp nanofiber achieved a wound closure rate of 67.7%. The addition of Cur helped achieve antibacterial, antioxidant, and mechanical properties while retaining biocompatibility with the tissue [101].

## 4. Zein

Zein is a naturally occurring protein polymer readily found in corn. Owing to its biocompatibility, degradability, flexibility, antibacterial, and oxidative properties, it has been widely used in biomedical applications such as tissue regeneration, drug delivery processes, and wound treatment [102,103].

To sustainably release sodium citrate or other drugs into the wound area, it is necessary to secure and protect the wound area and release the drugs. To achieve such functionality with added economic benefits, a method for developing nanofibrous dressings from corn zein was developed. Air jet spinning of the nanofibers enabled synthesis via a sustainable and economically beneficial drug release system that could be used to assist wound healing in patients with diabetes. Owing to significant experimentation performed to ensure that biocompatibility is not an issue, corn zein nanofibers have the potential to become reliable and sustainable drug release systems [92].

**Figure 10:**
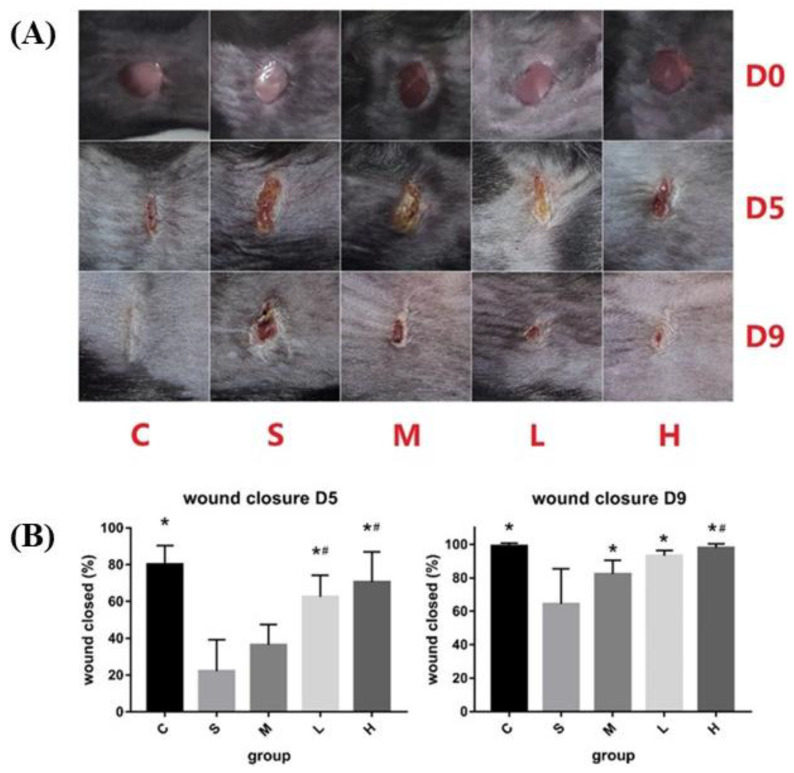
(**A**) Wound images at 0, 5, and 9 days (D0, D5, and D9). **H** Diabetic, 5% sesamol-CA/Zein composite nanofiber membrane; **S** Diabetic, no intervention; **M** Diabetic, CA/Zein blended nanofiber membrane; **L** Diabetic, 2% sesamol-CA/Zein composite nanofiber membrane; and **C** Normal. (**B**) Quantitative analysis of wound closure in diabetic mice at day 5 (D5) and day 9 (D9). * Statistically significant, *p* < 0.05 vs. group C, # Statistically significant, *p* < 0.05 vs. group S. Reproduced with permission from Ref. [93]. Copyright 2020, Elsevier.

The use of cellulose acetate (CA) with zein and sesamol produced CA/zein nanofiber membranes that acted as a delivery system for the controlled release of sesamol. To create the most effective membrane for delivery, it was determined that the ratio of CA to zein should be 12:8 to create the best membrane with a small diameter and uniform distribution. The created membrane exhibited an enhanced healing speed, as the membrane with sesamol promoted the creation of fibroblasts and inhibited chronic inflammation in the wounds (Figure 10) [93].

## 5. Conclusions and Future Perspectives

Diabetic wounds are complications in patients with diabetes that take a long time to resolve. These wounds produce high levels of inflammatory cytokines and ROS, which further damage normal cells and tissues. Furthermore, diabetic wounds exhibit repeated bacterial infections, owing to their hyperglycemic environments, which cause great suffering to patients and lead to a significant burden on health care systems. The development of wound dressings is necessary to protect and accelerate wound closure. Electrospun nanofibers are a better choice for diabetic wound closure because of their structural similarity to the ECM, which induces cell adhesion, proliferation, and migration, and subsequently, new tissue formation. Compared to conventional wound dressing materials, electrospun nanofiber dressing patches have high porosity and high moisture permeability, and they provide an effective barrier against external pathogen invasion [104,105]. They exhibit a remarkable potential for encapsulating and delivering active substances that promote wound healing. In a recent study, herbal compound (SRHC)-loaded electrospun MeGEL/PLLA dressing patches accelerated the wound healing rate compared to medical gauze in vivo [50]. Similarly, bioflavonoid (quercetin)-loaded zein-based nanofibers enhanced functional recovery in diabetic neuropathy [106].

Loading small molecules, drugs, and cells can accelerate the recovery process, but single drug loading may not achieve the desired outcome. Dual loading of bioactive agents, such as zinc nanoparticles and oregano essential oil, exhibits synergistic action in diabetic wound healing [106]. Combining multiple drugs or inorganic particles may provide a potential strategy for achieving the desired treatment. Recently, wound bandages with nanomaterials have shown great potential in improving diabetic wound healing. nCeO2-loaded poly(3-hydroxybutyrate-co-3-hydroxyvalerate) (PHBV) electrospun membranes enhance vascularization and cell proliferation in vivo [107]. Wound dressing containing silver nanoparticles (Acticoat^®^) are the first commercial product that may pave the way for novel approaches in diabetic wound healing [108]. Multilayer electrospun scaffolds may offer another method for improving diabetic wound healing [50]. Hydrogel fiber wound dressings, which have the advantage of good air permeability and liquid absorption, may provide an opportunity for the treatment of diabetic wounds.

Despite all the efforts taken to manage diabetic wounds, the intended therapeutic outcomes have not been achieved yet. Notably, diabetic wound healing studies rely heavily on in vivo animal experiments, and the healing effect is mostly determined by the healing time, whereas analyses of the diabetic wound healing process and tissue sections are limited. Furthermore, the lack of research on the biocompatibility and degradability of electrospun fibers makes safety evaluations difficult.

## Figures and Tables

**Table 1:** Synthetic polymers used for electrospun nanofibers to promote diabetic wound healing.

Polymer Used for Electrospun Fiber	Active Ingredient	Highlights	References
PCL/F-127		Modifiable size and depth enhanced angiogenesis and collagen deposition	[36]
PCL/Col	Bioactive glass nanoparticles	Improved cell attachment, proliferation and upregulated angiogenesis	[37]
PCL/Col1	Dimethyloxalylglycine	Regulation in release of DMOG and ability to stabilize HIF-1α levels and increase angiogenesis and re-epithelialization	[38]
PCL	Dimethyloxalylglycine	Increased wound closure rate, re-epithelization, and angiogenesis	[39]
Steviol glycosides polyurethane/PCL	-	Commendable mechanical characteristics, and high biocompatibility	[40]
PCL/Gum tragacanth	Curcumin	Improved antibacterial properties against *Staphylococcus aureus*, extended spectrum β lactamase, increased angiogenesis and wound closure	[41]
PCL/Col1	Calreticulin	Stimulated fibroblasts proliferation and allowed for keratinocyte migration	[42]
PCL/Col1	Melilotus officinalis extract	Improved re-epithelization and wound closure	[43]
PCL/Gelatin	Pioglitazone	Enhanced mechanical properties, cell proliferation, angiogenesis, and wound closure	[44]
Collagen/PLGA	Glucophage	Promoted wound healing rate, collagen levels, and downregulation of metalloproteinase 9	[45]
PLGA	Platelet-derived growth factor/Vancomycin or Gentamicin	Accelerated healing process, reduced phosphatase, and tensin content,	[46]
HA/PLGA	Epigallocatechin-3-O-gallate	Rapid decrease in wound area, improved re-epithelization and vasculiarzation	[47]
PLGA/Gelatin PDO/Gelatin	LL-37-mimic peptide W379	Accelerated wound healing, increased cell infiltration, vascularization, and re-epithelization	[48]
PLLA	Dimethyloxalylglycine	Improved vascularization, collagen deposition and re-epithelization Rapid stimulation of angiogenesis	[49]
Methacrylated gelatin/PLLA	*Salvia miltiorrhiza Bunge-Radix Puerariae*	Increased antibacterial properties against *S. aureus* and *E. coli*, Enhanced healing rate of the wound area	[50]
PLA/HA	Valsartan, ascorbic acid	Accelerated wound healing and re-epithelization	[51]
PLA/PVA	Cerium oxide nanoparticles	Improved in mechanical strength and biocompatibility. Enhanced wound healing rate through growth, adhesion and proliferation rate of fibroblasts	[52]
PLA/Gelatin	Epidermal growth factor	Antibacterial properties against *E. coli* and *S. aureus*, improved curative activities	[53]
PVA/chitosan	Desferrioxamine	Sustained release of desferrioxamine improved upregulation of Hif-1α and vascular growth factor. Increased cell proliferation and vessel formation	[54]
PLA/PVA	Gum tragacanth	Exhibited tissue repair and regeneration as well as collagen formation. Enhanced mechanical properties and young modulus.	[55]
PVA/Chitosan	-	Decreased in epidermal gap and rapid wound healing process	[56]
PVA/Chitosan	Zinc oxide	Enhanced antibacterial properties against *E. coli, P. aeruginosa, B. subtilis,* and *S. aureus* and increased rate of healing.	[57]
Pyrrolidine/m-PEG	Gentiopicroside, thymoquinone	Enhanced antibacterial effects against *P. aeruginosa*. Rapid wound closure rate through stimulation of cell growth, proliferation and inhibit inflammatory reaction	[58]
Polyvinylpyrrolidone	Pioglitazone	Inhibition of inflammation, management of skin condition through sustained release of pioglitazone	[59]

**Table 2:** Natural polymer used to create electrospun nanofibers to promote diabetic wound healing.

Polymer Used for Electrospun Fiber	Active Ingredient	Highlights	References
Gelatin/Arabinoxylan ferulate	Silver sulfadiazine	Enhanced mechanical properties and delivery of silver sulfadiazine over time enhanced antibacterial properties against *S. aureus*, *E. faecalis*, *P. aeruginosa*.	[88]
Gelatin/Bacterial cellulose	Metformin, glybenclamide	Sustained release of metformin or glybenclamide improved healing rate.	[89]
Gelatin/Poly-3-hydroxybutyrate	-	Improved fibroblasts cells adhesion and increased hair follicles and sweat glands	[90]
Silk fibroin/Poly-(L-lactide-co-caprolactone)	Huangbai Liniment	Improved antibacterial properties, mechanical properties, and wound closure rate. Downregulate the inflammatory factors	[91]
Corn zein	Sodium citrate	Sustained release of sodium citrate enhanced the healing rate, increased thermal and mechanical properties, and exhibited higher biocompatibility	[92]
Cellulose acetate/Zein	Sesamol	Inhibited chronic inflammation and promoted fibroblast proliferation	[93]
